# The assessment of local response using magnetic resonance imaging at 3- and 6-month post chemoradiotherapy in patients with anal cancer

**DOI:** 10.1007/s00330-016-4337-z

**Published:** 2016-04-18

**Authors:** Rohit Kochhar, Andrew G. Renehan, Damian Mullan, Bipasha Chakrabarty, Mark P. Saunders, Bernadette M. Carrington

**Affiliations:** 1 Department of Radiology, The Christie NHS Foundation Trust, Wilmslow Road, Manchester, M20 4BX UK; 2 Institute of Cancer Sciences, The University of Manchester, Manchester Academic Health Science Centre, Manchester, UK; 3 Department of Surgery, The Christie NHS Foundation Trust, Manchester, UK; 4 Department of Histopathology, The Christie NHS Foundation Trust, Manchester, UK; 5 Department of Clinical Oncology, The Christie NHS Foundation Trust, Manchester, UK

**Keywords:** Magnetic resonance imaging, Anus neoplasms, Carcinoma, squamous cell, Chemoradiotherapy, Tumour response

## Abstract

**Objectives:**

To assess the use of MRI-determined tumour regression grading (TRG) in local response assessment and detection of salvageable early local relapse after chemoradiotherapy (CRT) in patients with anal squamous cell carcinoma (ASCC).

**Methods:**

From a prospective database of patients with ASCC managed through a centralised multidisciplinary team, 74 patients who completed routine post-CRT 3- and 6-month MRIs (2009–2012) were reviewed. Two radiologists blinded to the outcomes consensus read and retrospectively assigned TRG scores [1 (complete response) to 5 (no response)] and related these to early local relapse (within 12 months) and disease-free survival (DFS).

**Results:**

Seven patients had early local relapse. TRG 1/2 scores at 3 and 6 months had a 100 % negative predictive value; TRG 4/5 scores at 6 months had a 100 % positive predictive value. All seven patients underwent salvage R0 resections. We identified a novel ‘tram-track’ sign on MRI in over half of patients, with an NPV for early local relapse of 83 % at 6 months. No imaging characteristic or TRG score independently prognosticated for late relapse or 3-year DFS.

**Conclusions:**

Post-CRT 3- and 6-month MRI-determined TRG scores predicted salvageable R0 early local relapses in patients with ASCC, challenging current clinical guidelines.

***Key Points*:**

• *Post-chemoradiotherapy MRI (3 and 6 months) helps local response assessment in ASCC*.

• *The MRI-TRG system can be used reproducibly in patients with ASCC*.

• *The TRG system facilitates patient selection for examination under anaesthesia and biopsy*.

• *The use of MRI-TRG predicts for detection of salvageable early local relapses*.

• *The TRG system allows for a standardised follow-up pathway*.

**Electronic supplementary material:**

The online version of this article (doi:10.1007/s00330-016-4337-z) contains supplementary material, which is available to authorized users.

## Introduction

For patients with anal squamous cell carcinoma (ASCC), trials performed in the 1990s [1, 2] established chemoradiotherapy (CRT) as standard initial treatment. Despite these improvements, recent trials [3, 4] continue to observe early local relapse, within the first 12 months, of approximately 10 % after CRT [4], and total local relapse rates (additionally including late local relapses after an initial disease-free period) between 18 % and 25 % [3, 5]. Salvage radical surgery, typically in the form of abdomino-perineal resection, offers a second chance for cure in these patients with R0 resection (microscopic negative margins) [6]. However, many institutional series report post-salvage resection positive margin (R1/R2) rates between 16 % and 20 % [7–10], and as high as 32 % in population registries [11]. For patients with R1/R2 resections, post-salvage survival rates are dismal – typically zero survival at 3–5 years [5, 7, 10, 11] – rates equivalent to those observed in patients with local relapse not undergoing salvage surgery [5], thus questioning patient benefit from such radical surgery. Against the above background, follow-up should be directed towards the early detection of salvageable local relapse. Here, we posit (contrary to current guidelines, below) that magnetic resonance imaging (MRI), the imaging modality of choice for locoregional staging [12–15], has a role in post-CRT assessment of treatment response and detection of salvageable local relapse, and specifically test this hypothesis for early local relapse in the first 12 months (when 60 % of all local relapses occur [5]).

Guidelines from the European Society for Medical Oncology (ESMO) recommend clinical evaluation for complete response at 8 weeks post-CRT and then 3- to 6-monthly thereafter for a 2-year period, stating that “MRI can capture and document response, but no individual MRI feature appears predictive of eventual outcome” [14]. The ESMO guidelines endorse a ‘watchful wait’ approach, stating that “partial regression can be managed by close follow-up, to confirm that (a delayed) complete regression takes place, which may take 6 months”. The US National Comprehensive Cancer Network (NCCN) Clinical Practice Guidelines make no specific reference to the role of MRI in post-CRT surveillance [16], and the American Society of Colon and Rectal Surgeons guidance [17] does not endorse a role for MRI post-CRT surveillance, citing evidence that MRI is not a predictor of clinical response in early (6–8 weeks) follow-up of CRT [13] and that longer term (6–12 months) MRI evaluation can demonstrate changes in tumour size and a reduction/stabilisation of signal intensity, but correlates only modestly with outcome [13]. However, both cited series supporting this recommendation were limited to small sample sizes (35 [13] and 15 [18] patients, respectively) and optimal timing of MRI post-CRT remains unclear [19].

At our centralised anal cancer multi-disciplinary team (MDT) we have long had a proactive (rather than a watchful wait) approach to the detection and surgical intervention for early local failure after CRT in patients with ASCC [5, 6]. Since 2009, this protocol has included routine post-treatment MRI assessment at 3 and 6 months. The primary aim of this study was to determine the use of this MRI-inclusive protocol to assess tumour response and the detection of salvageable early local failure after CRT. To this end, we extended the use of the MRI tumour regression grading (TRG) system used in other tumour settings (such as oesophageal [20] and rectal cancer [21, 22]) and established good reproducibility. As a secondary aim, we identified and described a novel ‘tram-track sign’ as a putative indicator of complete local tumour response, and evaluated its significance.

## Materials and methods

### Patients and treatment

Between 2009 and 2012, 118 patients were managed consecutively through our centralised MDT for patients with anal malignancies. To investigate the relationship between MRI changes from baseline to 3 and 6 months post-CRT with early outcomes, patients who satisfied the following criteria were included: (1) histological confirmation of ASCC without metastatic disease at baseline; (2) the presence of a demonstrable anal lesion on baseline staging MRI; (3) standard treatment with CRT including an inguinal radiation boost where indicated; (4) follow-up MRI scans at 3 and 6 months post-CRT (±2 weeks on either side); (5) histological correlation with examination under anaesthesia (EUA) and biopsy or at least 2 years’ follow-up post-CRT. This approach mimicked a per-protocol analysis of routine 3- and 6-month MRI surveillance. Seventy-four patients met these criteria.

The following patients were excluded: no demonstrable lesion on staging MRI, 15; inadequate follow-up, 15 (6-month post-treatment MRI not done in 12 and delayed timing of post-treatment scans in three); recurrent ASCC, four; ectopic or multifocal SCC, five; other histological subtypes, five. This retrospective study was approved by the institutional audit and service improvement committee and informed consent was waived.

All patients received CRT using the UK National Anal Cancer Trial (ACT II) protocol [4]. Radiotherapy of 50.4 Gy was delivered in 28 daily fractions (F) over 5.5 weeks with a two-phase technique. Phase 1 delivered 30.6 Gy (17 F) using non-conformal rectangular parallel-opposed fields aiming to treat all pelvic nodes (except the common iliac); phase 2 was conformally planned using CT images to deliver 19.8 Gy (11 F) over 15 days treating the primary tumour and the whole anal canal with a 3-cm margin around the macroscopic tumour. Patients received fluorouracil 1,000 mg/m^2^ per day on days 1–4 (week 1) and 29–32 (week 5) by continuous 24-h intravenous infusion with radiotherapy and 12 mg/m^2^ of mitomycin as an intravenous bolus on day 1 only.

### Magnetic resonance imaging (MRI) and staging

All baseline staging MRI scans were performed on a 1.5-T MRI unit employing a pelvic phased-array body coil. The acquisition protocol is detailed in Table 1 (Supplementary file). The same protocol was performed for post-CRT MRI. Multiparametric imaging with dynamic post-contrast sequences (DCE) and diffusion-weighted imaging (DWI) are not routinely used in our clinical practice as these are still considered research modalities [23]. The baseline and post-CRT MRI scans were reviewed, blinded to outcome, by two radiologists with 25 and 8 years’ experience, respectively, in reading pelvic MRI, assigning final TRG scores by consensus.

The following primary tumour features were assessed: size (the maximum diameter in any plane measured on the high resolution T2W image to the nearest millimetre); position (anal canal, anal margin); circumferential tumour extent (position on clock); subjective T2W signal intensity (high–similar to fluid; low–similar to muscle and intermediate); infiltration of adjacent organs (vagina, prostate); trans-sphincteric extension into the ischioanal/ ischiorectal fossa; and the presence of perianal fistulas or abscesses. Regional and metastatic lymph node involvement was reported using accepted size criteria [24] and signal intensity characteristics. Nodal sites were defined and tumour stage was recorded using the AJCC TNM system, seventh Edition [25].

### MRI and treatment response evaluation

Tumour response was evaluated on the 3- and 6-month post-treatment MRI scans. Residual tumour size was recorded and if there was no suspicious residual focus, then the tumour was considered to have completely responded. Regression or enlargement of malignant nodes seen on staging MRI or any new suspicious lymph node enlargement was also recorded. Development of metastatic disease on follow-up was recorded. We also correlated primary tumour stage with local tumour response.

Tumours response was additionally scored 1–5, using the Mandard tumour regression grading (TRG) system (Table 1), similar to that used after CRT for rectal cancer [21, 22]. TRG scores were given based on careful analysis of the signal intensity changes on the post-CRT MRI scans and correlation with baseline MRI to match with the original tumour location and characteristics.

**Table 1:** Tumour regression grading scores for anal cancer on post-treatment magnetic resonance imaging

	Description
Grade 1	Complete response with no evidence of tumour and normal appearances of the anus
Grade 2	Excellent response with only low signal post treatment fibrotic change and no evidence of tumour
Grade 3	Moderate response with indeterminate heterogeneous signal intensity at the tumour site
Grade 4	Minimal response with reduction in size but evidence of intermediate tumour signal in keeping with residual disease
Grade 5	No response of the primary tumour or frank tumour progression

**Fig. 1:**
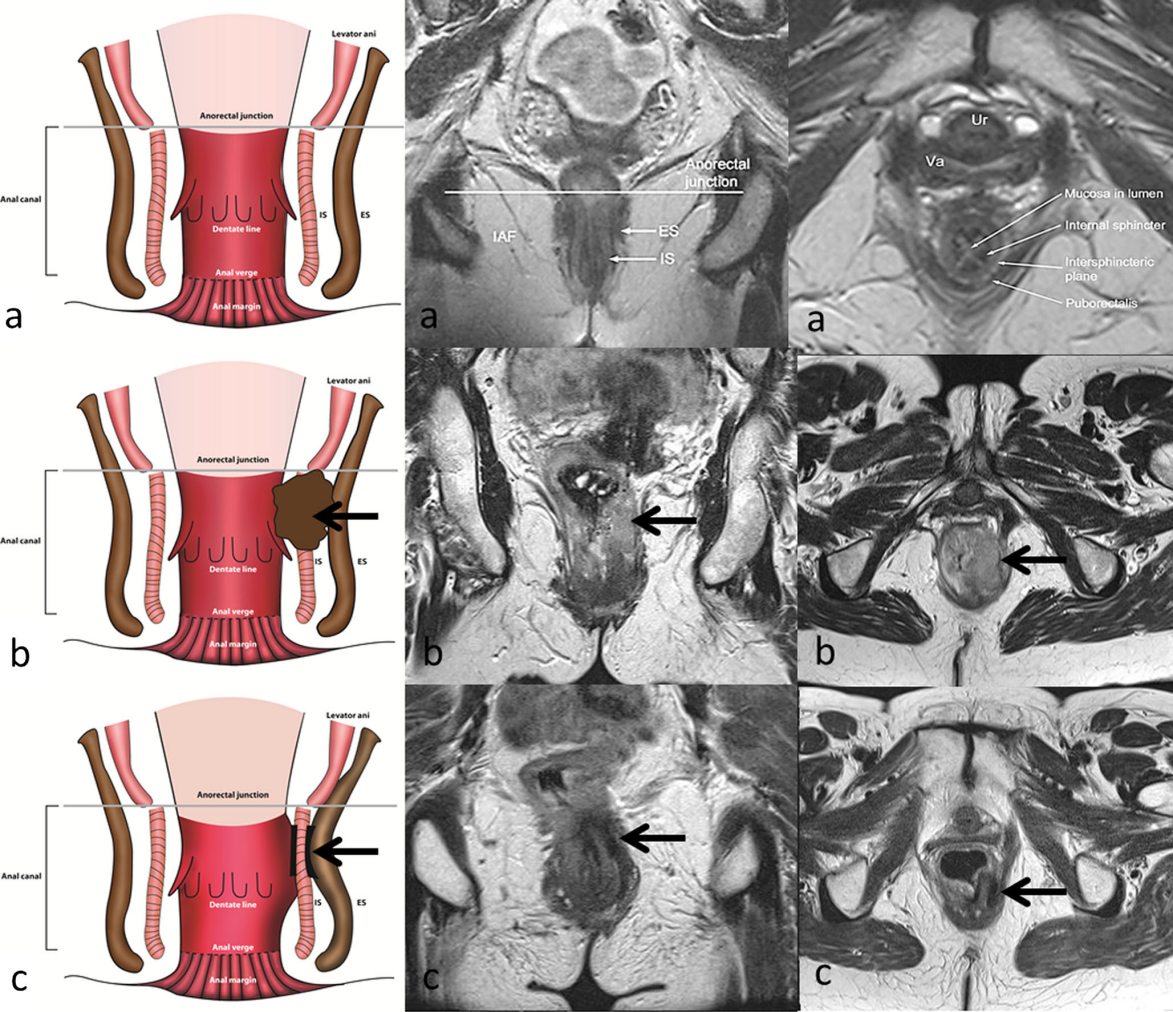
Demonstrative figure showing the normal anal anatomy in row ‘**a**’ (line diagram representation in left column, high resolution T2W MR in the coronal plane in mid column and in the axial plane in the right hand column). Row ‘**b**’ demonstrates an upper anal canal tumour involving the left internal sphincter extending from the 2 o’clock to 6 o’clock position (arrows). Post-CRT appearances in row ‘**c**’ showing the tram track sign as parallel bands of low signal along the inner and outer margins of the left internal sphincter at the site of original tumour (arrows)

In patients with tumours involving the anal canal, the presence of a novel ‘tram track’ sign was recorded on the post CRT MRI scans. This was defined as parallel linear low signal at the inner and outer margin of the internal sphincter, at the site of the original tumour (Fig. 1a–c). This sign was analysed as a marker of complete local tumour response.

### Follow-up and outcomes

Clinical assessment was first performed in all patients at 6 weeks after completion of CRT and again at clinical visits paralleling the 3- and 6-month scans. Based on the findings of clinical assessment supplemented with the MRI findings, patients were subjected to EUA with or without biopsy to confirm or exclude local disease relapse.

### Statistical analysis

All computations were performed using Stata™ 12.0 (College Station, TX, USA). TRG scores were correlated with the subsequent development of early local relapse (defined as within 12 months following CRT completion) to estimate performance characteristics (sensitivity, specificity, positive predictive value (PPV) and negative predictive value (NPV)) by categorising TRG scores as follows: patients with TRG grades 1 or 2 were grouped together as complete responders and those with grades 4 or 5 as non-responders. TRG grade 3 was the indeterminate group.

We determined actuarial rates for local relapse and disease-free survival (DFS) using standard Kaplan-Meier curves. Events in the DFS analyses were: any local relapse, distant metastases and death from any cause. Actuarial rates for local relapse were from date of completion of CRT; those for DFS were from start date of initial treatment. We tested a number of patient, tumour and imaging characteristics against DFS and estimated effects sizes (expressed as hazard ratios (HRs) and their 95 % confidence intervals (CIs)) using Cox models. We derived the final models using a seven-step approach as we have previously described [26], exploring for confounding interactions, collinearity problems, predictive accuracy and calibration.

Reproducibility (inter-reader agreement) was assessed by calculating the *k* (kappa) values, including a weighting option to weight for clinically important disagreements, and interpreted as follows: less than 0.20, poor agreement; 0.21–0.40, fair agreement; 0.41–0.60, moderate agreement; 0.61–0.80, good agreement; and 0.81–1.00, excellent agreement [27].

## Results

### Histology

All 74 patients had histologically confirmed anal SCC. Of these, ten were well differentiated, 25 were moderately differentiated and 14 were poorly differentiated SCCs, and in 25 patients this information was not available.

### Baseline characteristics

The baseline characteristics of the 74 patients with histologically confirmed ASCC are listed in Table 2. In common with most treatment series from European populations [28], females accounted for approximately 55 % of patients. There were five patients with confirmed HIV positivity. Only five tumours (7 %) were fully confined to the anal margin, the remainder arising solely in the anal canal or overlapping the anal canal and margin. Approximately two-thirds of these were stage T2, approximately half were node positive.

**Table 2:** Baseline characteristics in 74 patients with anal squamous cell carcinoma (ASCC)

	Characteristic
Median age (range) year	60 (33 to 86)
Male : female	34 : 40
Median tumour size (range) cm	4.1 (1.1 to 11.8)
Anatomical location	
Canal	58 (78)
Margin	5 (7)
Both canal and margin	11 (15)
Circumferential involvement	
Entire anal circumference	18 (24)
Greater than 50 % circumferential	17 (23)
Less than 50 % circumferential	39 (53)
Tumour extension into lower rectum	32 (50)
Transphincteric extension	13 (18)
Involvement of adjacent organs	10* (14)
T stage	
T1	4 (5)
T2	50 (67)
T3	10 (14)
T4	10 (14)
Nodal stage	
N0	38 (51)
N1	14 (19)
N2	7 (10)
N3	15 (20)

Note: Values in parentheses are percentages unless otherwise stated

*Vagina, 8; prostate, 2

Other features: perianal fistula, 4; abscess, 1

### Follow-up and local relapse

As per inclusion criteria for analysis, all patients had a minimum of two post-treatment MRI scans. Clinical follow-up of over 2 years after CRT was available in 67 of the 74 patients. Of the seven patients who did not have a follow-up of over 2 years, six had died in the interim (four from unrelated causes and two due to metastatic disease from anal cancer) and one patient did not attend any further follow-up. During the first 12 months, EUA and biopsy was performed in 32 of the 74 patients.

**Fig. 2:**
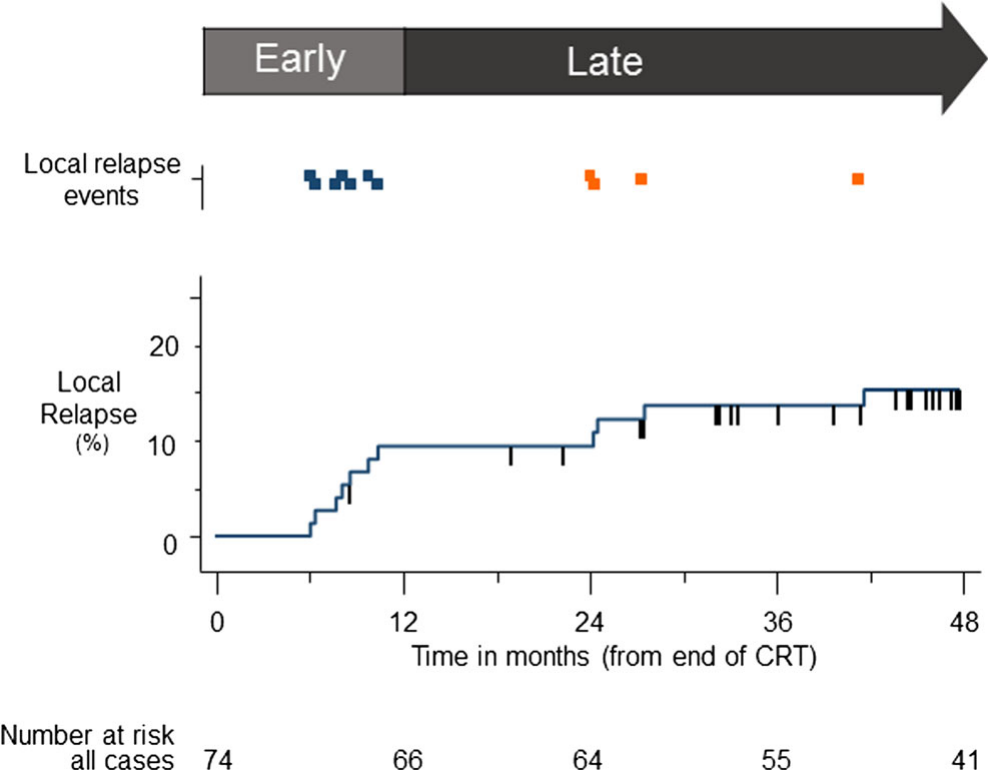
Time to event plot for local relapses after chemoradiotherapy (CRT) shown by early (within 12 months) and late (after 12 months) relapses. Short vertical hashes represent censored events. Markers in upper panel indicate local relapse events: blue, early relapses; orange, late relapses

With a median follow-up of 52 (range 9–72) months after completion of CRT, there were 11 (15 %) local relapses; seven (9 %) were early relapses (within the first 12 months) and four were late relapses (in this series, all late local relapses were after 24 months) (Fig. 2). The 3-year actuarial rate of local relapse was 14 % (95 % CI: 8–24). Among the seven patients with early local relapse, there was a relationship with increasing T-stage as follows: T1: 0/4; T2: 4/50 (8 %); T3: 1/10 (10 %); and T4: 2/10 (20 %).

### Post-treatment MRI findings

Following treatment, there was a reduction in tumour size at 3 months (mean reduction 28.79 %, range 0–100) and 6 months (mean reduction 81.39 %, range 0–100). There was a subjective reduction in signal intensity of the primary tumour compared to baseline in 69 % (51/74) of patients on the 3-month scan and in 84 % (62/74) of patients on the 6-month scan. Nodal disease was down staged in 33 of the 36 patients, with N0 status in 32 of these patients.

### Tumour regression grading (TRG) score

**Fig. 3:**
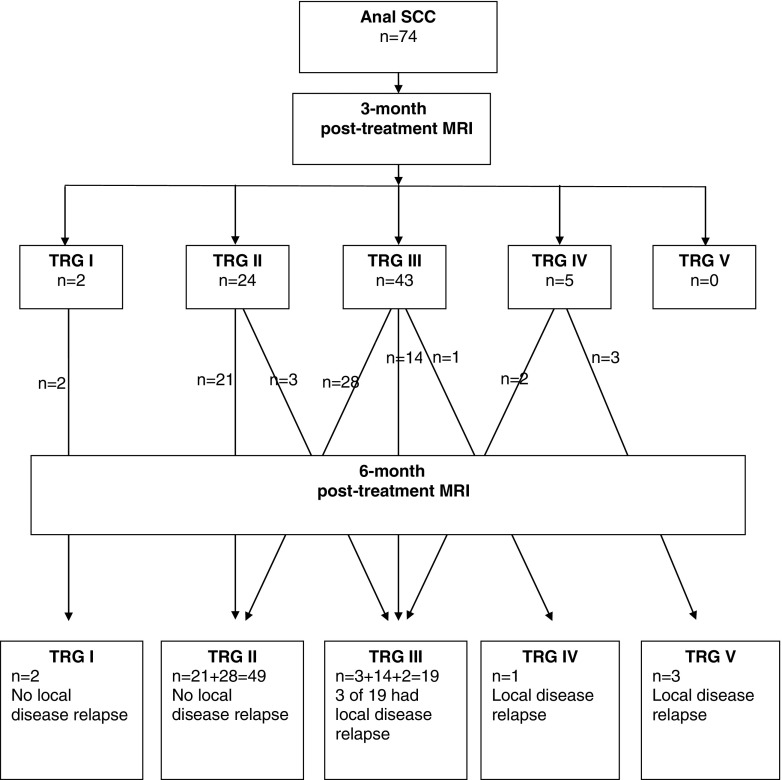
Tumour regression grading (TRG) scores on post-treatment magnetic resonance imaging (MRI) correlated with local disease relapse. *SCC* squamous cell carcinoma

**Fig. 4:**
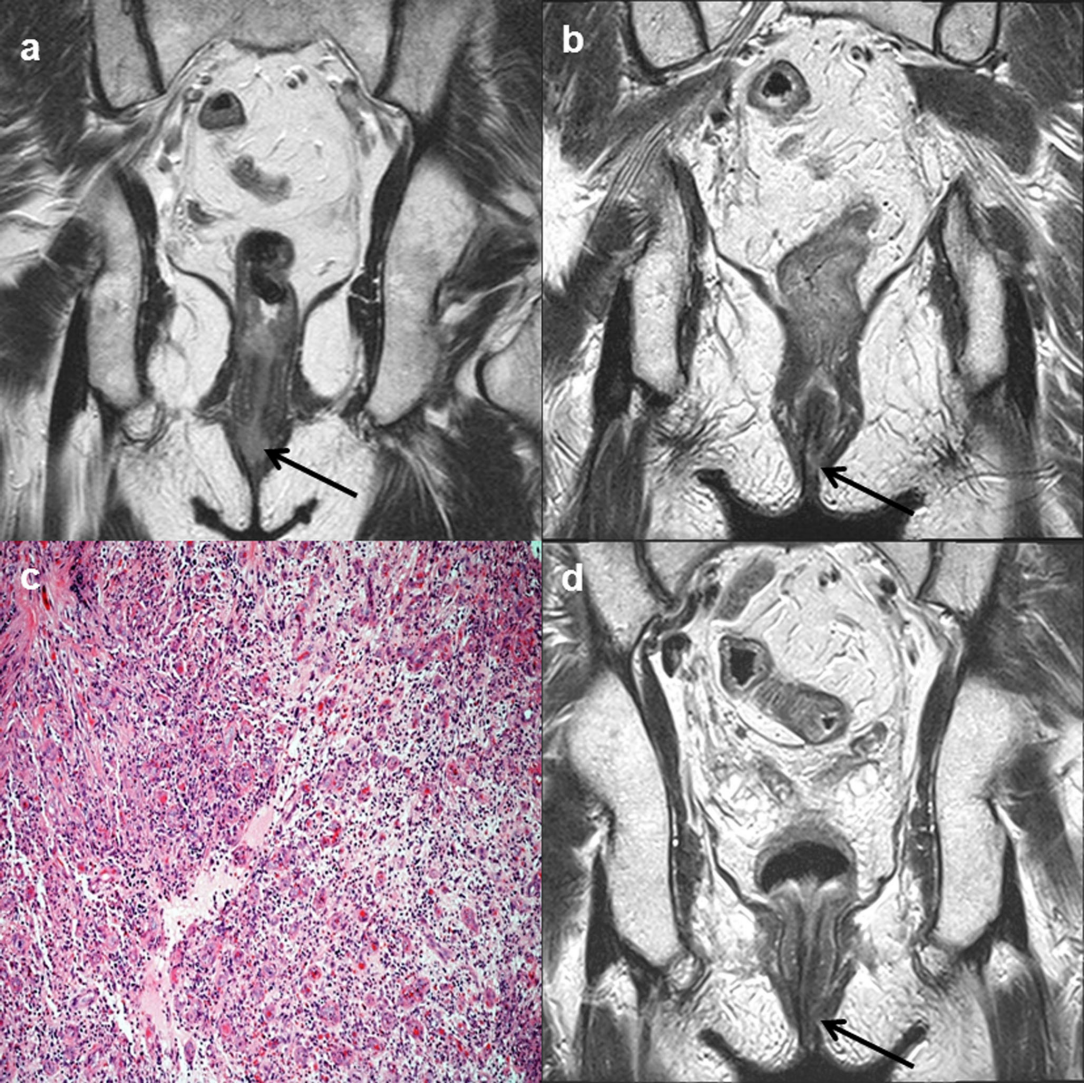
High resolution coronal T2-weighted images (**a**, **b**, **d**). Baseline magnetic resonance image (MRI) (**a**), showing an intermediate signal intensity tumour (arrow) in the lower anal canal extending to the verge. The 3-month post-chemoradiotherapy (CRT) MRI (**b**) shows response to treatment but with mixed low and high signal areas at site of original tumour (arrow), this was considerate indeterminate for residual disease versus inflammation, tumour regression grading (TRG) score 3. Photomicrograph with H & E stain and 20X magnification (**c**), showing partially organising and inflamed granulation tissue with no viable residual tumour, this correlates with TRG 3 on the post-CRT MRI. The 6-month post CRT MRI (**d**) now shows improvement in appearances with low signal change and no evidence of any suspicious intermediate signal indicating the previously noted changes due to inflammation had resolved, downgrading the TRG score to 2

**Fig. 5:**
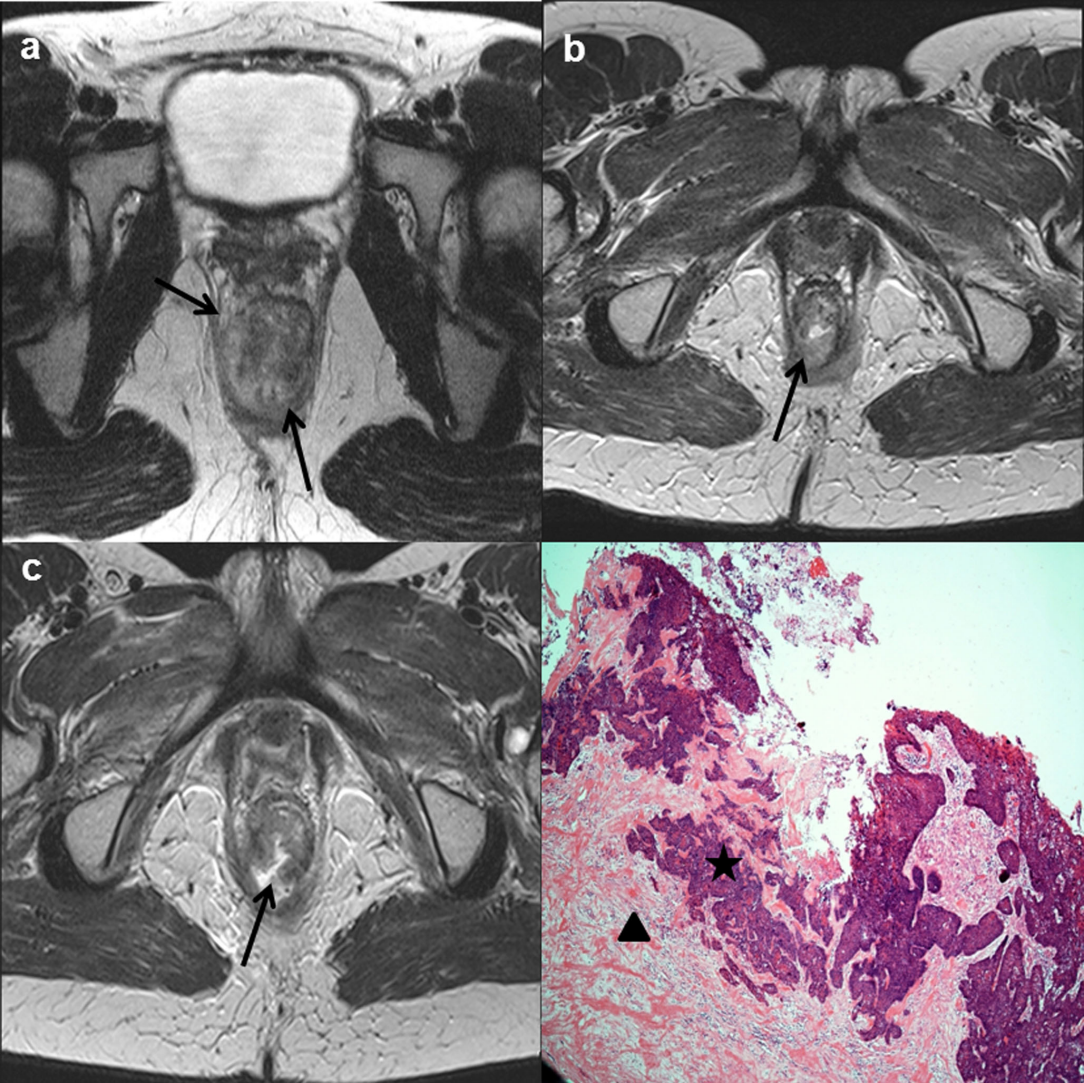
High resolution axial T2-weighted images (**a**-**c**). Baseline magnetic resonance image (MRI) (**a**), showing an intermediate signal intensity tumour (arrows) in the anal canal extending from the 5 o’clock to 11 o’clock position. The 3-month post-chemoradiotherapy (CRT) MRI (**b**), shows decrease in size but residual intermediate signal remains of concern for tumour (arrow), tumour regression grading (TRG) score 4. The 6-month post CRT MRI (**c**) shows interval progression with central cavitation of the suspected residual tumour (arrow), TRG score 5. Photomicrograph with H & E stain and 4X magnification showing residual viable invasive squamous cell carcinoma (star shape) associated with some underlying stromal hyalinisation (triangle shape) in response to prior radiotherapy, these histological features correlate with Grades 4 to 5 on the post-CRT MRI. The patient subsequently underwent radical surgery with flap reconstruction

The TRG scores on the post-treatment MRI at 3 and 6 months, temporal score change and correlation with local relapse are summarised in Fig. 3. All seven patients underwent salvage R0 resections. High-resolution images and case studies of the TRG scoring for anal cancers are shown in Figs. 4 and 5 (Figs. 1 and 2 are Supplementary files). Performance characteristics by various TRG models for 3- and 6-month TRG scores and changes with time are shown in Table 2 (Supplementary file). All patients who demonstrated TRG 1 and 2 on 3- and 6-month post-CRT MRI scans showed no evidence of early local relapse. This gave a negative predictive value of 100 % for early local relapse. On the 6-month MRI, all four patients with TRG 4 and 5 had histological confirmation of residual disease on EUA. This gave a positive predictive value of 100 % for residual disease. Table 2 (Supplementary file) also shows four patterns of TRG changes between 3- and 6-month scans: TRG1/2 stable (35 %); TRG3, further regression (38 %); TRG3 stable (22 %); and progression (5 % or four cases). However, this categorisation did not appear to add additional discrimination above and beyond those models for TRG scored at 3 and 6 months and simply compared with baseline scans.

### Tram-track sign

We identified a novel post-treatment ‘tram-track’ sign on MRI. This was a parallel linear low signal at the inner and outer margin of the internal sphincter, at the site of the original tumour. This is due to a band-like fibrotic treatment response in the muscularis sub-mucosa and bands of fibrosis in and between the smooth muscle of the internal sphincter, and the skeletal muscle of the external sphincter which is relatively spared (histology of the same patient as in Fig. 1 is shown in Supplementary Fig. 1d).

We tested the hypothesis that this sign is a marker of complete local tumour response. Of the 69 patients with anal canal tumours, the tram-track sign was seen in 48 % (33/69) on the 3-month scan, and in 57 % (39/69) on the 6-month MRI scan. Of these 39 patients, only one developed early local disease relapse. At presentation this patient had a large tumour involving both the canal and the margin, and early relapsed disease occurred in the anal margin separate from the tram-track sign in the anal canal.

### Disease-free survival

During follow-up, in addition to local relapses, metastatic disease was detected in seven patients. For all patients, the 1-year and 3-year DFS rates were 85 % (95 % CI: 75–92) and 72 % (95 % CI: 60–80). We tested various patient, tumour and imaging characteristics against DFS (Table 3, Supplementary file). By univariate analyses, the following factors were significant: performance status; T-stage; circumferential involvement, trans-sphincteric extension, involvement of adjacent organs, TRG 4/5 at 3 months and 6 months. By multivariate modelling, testing for independence, only the following remained significant: performance status and T-stage (borderline). No imaging characteristic or TRG score independently prognosticated for late relapse or 3-year DFS.

### Reproducibility of the tumour regression grading

The inter-rater kappa values for the TRG scores on the 3- and 6-month scans were 0.61 and 0.76, respectively, demonstrating good inter-rater agreement in accordance with the priori criteria [27].

## Discussion

The role and timing of MRI for post-CRT response assessment in patients with ASCC is not clear. Our study has shown that MRI performed at 3 and 6 months post-CRT, interpreted using the TRG system, is helpful in assessing and categorising local response and in guiding further management.

Traditionally clinical response at 6–8 weeks post-CRT is considered a predictor of locoregional control [29]. However, it may take 3–6 months for complete tumour resolution to occur. In the Anal Cancer Trial (ACT II) the optimum time to assess complete clinical response was reported as 26 weeks based on digital rectal examination and abdominopelvic CT; MRI and positron emission tomography (PET) were not considered essential [30]. Although clinical assessment is vital, it is important to be able to assess response non-invasively.

Only a few single-centre, small series have investigated the role of MRI to image response in ASCC [19]. A study from the London Royal Marsden investigators of 15 patients suggested that size involution is most evident at 6 months post-treatment compared with the immediate post-treatment stage where inflammation is superimposed on treated disease [18]. A study of 35 patients, from the London Mount Vernon investigators, showed that early assessment of response by MRI at 6–8 weeks was unhelpful in predicting future clinical outcome [13]. The present study similarly found a lack of association between imaging characteristics and DFS. Due to the rarity of ASCC and lack of bigger series, the optimal timing for therapy assessment with MRI has not been reported to date [19]. From the above studies it was anticipated that if MRI assessment is done within 6–8 weeks post-CRT, not all tumours will have achieved complete response; we therefore performed MRI assessment at two time points: 3 months and 6 months post-completion of CRT.

The accurate and reproducible interpretation of post-CRT MRI scans in patients with anal SCC is challenging, likely due to the complexity of anatomy, relative lack of experience due to rarity of this disease and difficulty in differentiating tumour signal from post treatment changes. Previous studies have shown that tumour size change and signal intensity change post-CRT are not predictive of clinical outcome [13]. Tumour regression grading (TRG) is known to be a better predictor of outcome after treatment than T stage in rectal cancers [31]. We therefore used the Mandard TRG system (Table 1), modified from Dworak et al. [32], to grade tumour response on MRI and guide further follow-up.

**Fig. 6:**
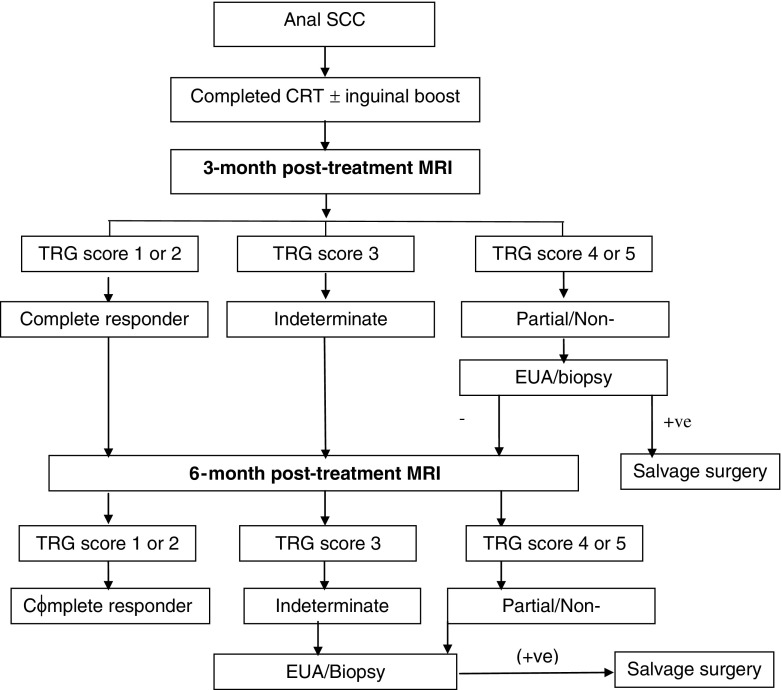
Proposed patient follow-up pathway based on post-chemoradiotherapy (CRT) magnetic resonance imaging (MRI) tumour regression grading (TRG) system. *SCC* squamous cell carcinoma

A clinical decision tree for the patient management and follow-up pathway based on this TRG scoring system is proposed in Fig. 6, but is based on the findings of this study alone and would need validation with more data from prospective multicentre trials.

There are some limitations to our study. Firstly, this is a retrospective study which might lead to recall bias during the interpretation of imaging. To avoid such bias, clinical and pathological outcomes were unavailable to the reviewers. There is a selection bias as patients with small anal cancers that had been removed by excision biopsy were excluded. Third, despite the relatively large denominator, the number of events of interest (early local relapse) was small and estimates of performance characteristics and associations with survival had wide CIs.

The study has several strengths. To our knowledge, this is the first time that TRG scoring has been reported in the context of anal cancer. We demonstrated that this scoring system is reproducible in this setting. Second, we comprehensively characterised imaging features on pre-treatment MRI and found some (e.g. trans-sphincteric extension) to prognosticate for DFS, but independence was lost in the multivariate modelling, suggesting that these imaging features are in part captured by T staging. Third, we described a novel ‘tram-track sign’ on imaging, which, when present, predicted for complete local response. However, this sign is not applicable to anal margin tumours and does not prognosticate for survival.

### Future research

There are a number of unanswered questions. First, there are other MRI modalities, such as dynamic post-contrast sequences (DCE) and diffusion-weighted imaging (DWI) [23], and these are currently under investigation as additional assessment for local response. Second, volumetric assessment of tumour and/or nodal volume as evaluated in other malignancies such as rectal cancer [33], might have a role in tumour response prediction in ASCC, and is the subject of ongoing study. Third, the addition of PET to MRI might add further refinement. A recent meta-analysis [34], albeit with small numbers of studies, suggests that PET might correctly assess complete response in over 80 % of cases. Finally, while we focused on the seven early local relapses in the current analysis, there were an additional four patients with late relapse. There were no clear predictors for these relapses. This now needs to be an area of research to better inform surveillance programmes beyond 12 months. Candidate tissue biomarkers include lack of p16 expression, which has incomplete concordance with lack of detection of HPV16 [35], and predicts for radio-resistance [36].

## Conclusion

In patients with anal cancers follow-up MRI performed 3 and 6 months post-chemoradiotherapy and scored using the tumour regression grading system allowed reproducible assessment of local tumour response and predicted for early local relapse. The ‘tram-track’ sign was a predictive indicator of local response in anal canal tumours. These findings challenge current clinical guidelines.

## Electronic supplementary material

Below is the link to the electronic supplementary material.

Table 1 (DOC 31 kb)

Table 2 (DOC 46 kb)

Table 3 (DOC 85 kb)

Fig. 1 High resolution coronal T2-weighted images (**a-c**). Staging MRI (**a**), showing an intermediate signal intensity tumour (arrow) in the anal canal involving the left internal sphincter. The 3-month (**b**) and 6-month post-CRT MRI (**c**), showing parallel linear low signal at the inner and outer margin of the left internal sphincter, at the site of the original tumour in keeping with a tram track sign (arrows) with no suspicious residual intermediate signal. Please note high signal mucosal oedema (block arrow) at the anorectal junction on the 3-month post CRT MRI. Photomicrograph with Haematoxylin and Eosin (H & E) stain and 20X magnification of a section of the outer portion of the internal anal sphincter (**d**), showing bands of stromal fibrosis in response to prior radiotherapy (triangle shapes), alternating with bands of residual atrophic skeletal muscle bundles (star shapes) which accounts for the tram track appearance. No residual invasive tumour seen. These histological features correlate with TRG 2 on post-CRT MRI (GIF 282 kb)

High resolution image (TIF 3981 kb)

Fig. 2 High resolution coronal T2-weighted images (**a-c**). Baseline MRI (**a**), showing an intermediate signal intensity tumour (arrow) in the anal canal. The 3-month post CRT MRI (**b**) shows response to treatment but with suspicious intermediate signal at original tumour site (arrow), TRG score 4. EUA biopsy performed was however negative for disease. Note extensive high signal post treatment oedema at the anorectal junction. The 6-month post CRT MRI (**c**), now shows improvement in appearances with predominantly low signal change (arrow) downgrading the TRG score to 2 (GIF 98 kb)

High resolution image (TIF 4493 kb)

## Abbreviations

ASCC: Anal squamous cell carcinoma
CRT: Chemoradiotherapy
ESMO: European Society for Medical Oncology
EUA: Examination under anaesthesia
NCCN: National Comprehensive Cancer Network
TRG: Tumour regression grading
