# Supplementary material for: The assessment of local response using magnetic resonance imaging at 3- and 6-month post chemoradiotherapy in patients with anal cancer
Source: Eur Radiol. 2016 Apr 18;27(2):607–17. doi: 10.1007/s00330-016-4337-z (PMC5209434; doi:10.1007/s00330-016-4337-z)
Supplement: Supplementary file 1 — (DOC 31 kb) [file 330_2016_4337_MOESM1_ESM.doc]

**Table 1 MRI protocol for imaging anal cancer**

|  | **Sequences** | **Acquisition Parameters** | **Comment** |
| --- | --- | --- | --- |
|  |  |  |  |
| Tumour | HR T2 sagittal TSE | TR/TE 5390/100 ms; NEX 3; ST 3 mm; FOV 200 | Sacral promontory down to cover anal margin |
| Tumour | HR T2 axial TSE | TR/TE 5030/100 ms; NEX 2; ST 3 mm; FOV 200 | Perpendicular to long axis of anal canal, x 2 overlapping blocks |
| Tumour | HR T2 coronal TSE | TR/TE 6500/137 ms; NEX 2; ST 3 mm; FOV 200 | Parallel to long axis of anal canal |
|  |  |  |  |
| Tumour | STIR axial  STIR coronal / sagittal | TR/TE 9840/97 ms; NEX 1; ST 3mm  TR/TE 5960/68 ms; NEX 2; ST 3mm | Only if suspicion of fistula |
| Abdomen / Pelvis | T1 axial SE | TR/TE 400/12 ms; NEX 1, ST 5mm; FOV 380 | 2/3 overlapping blocks, renal hila to symphysis |
| Abdomen / pelvis | T1 coronal SE | TR/TE 668/19 ms; NEX 2, ST 6mm; FOV 490 | Diaphragm to symphysis pubis |
|  |  |  |  |

HR= high resolution; NEX=number of excitations; FOV=field of view; ST=slice thickness; SS-EPI= steady state echo planar imaging
