# Supplementary material for: The assessment of local response using magnetic resonance imaging at 3- and 6-month post chemoradiotherapy in patients with anal cancer
Source: Eur Radiol. 2016 Apr 18;27(2):607–17. doi: 10.1007/s00330-016-4337-z (PMC5209434; doi:10.1007/s00330-016-4337-z)
Supplement: Supplementary file 2 — (DOC 46 kb) [file 330_2016_4337_MOESM2_ESM.doc]

**Table 2** Performance characteristics by various TRG models for 3- and 6-month TRG scores and changes with time

|  |  |  |  |  |  | **Performance characteristics** | | | |
| --- | --- | --- | --- | --- | --- | --- | --- | --- | --- |
|  | **n (%)** | **TP** | **FP** | **FN** | **TN** | Sensitivity  (95% CIs) | Specificity  (95% CIs) | PPV  (95% CIs) | NPV  (95% CIs) |
| **TGR categories at 3-month MRI*** |  |  |  |  |  |  |  |  |  |
| Model 3 (TRG 3/4/5 versus TRG 1/2) |  | 7 | 41 | 0 | 26 | 100 (59-100) | 39 (27-52) | 15 (6-28) | 100 (87-100) |
| Model 4 (TRG 4/5 versus TRG 1/2/3) |  | 3 | 2 | 4 | 65 | 43 (9-82) | 97 (90-100) | 60 (15-95) | 94 (86-98) |
| **TGR categories at 6-month MRI*** |  |  |  |  |  |  |  |  |  |
| Model 3 (TRG 3/4/5 versus TRG 1/2) |  | 7 | 16 | 0 | 51 | 100 (59-100) | 76 (64-86) | 30 (13-53) | 100 (93-100) |
| Model 4 (TRG 4/5 versus TRG 1/2/3) |  | 4 | 0 | 3 | 67 | 57 (18-90) | 100 (95-100) | 100 (40-100) | 96 (88-99) |
| **Changes in TGR from 3- to 6-month MRI** |  |  |  |  |  |  |  |  |  |
| Pattern 1: TRG 1/2 at 3 months | 26 (35) |  |  |  |  |  |  |  |  |
| Pattern 2: TRG 3 at 3 months → TRG 2 at 6 months | 28 (38) |  |  |  |  |  |  |  |  |
| Pattern 3: TRG 3/4 at 3 months → TRG 3 at 6 months | 16 (22) |  |  |  |  |  |  |  |  |
| Pattern 4: TRG 3/4 at 3 months → TRG 4/5 at 6 months | 4 (5) |  |  |  |  |  |  |  |  |
| Model 5 (Pattern 3/4 versus Pattern 1/2) |  | 7 | 13 | 0 | 54 | 100 (59-100) | 81 (69-89) | 35 (15-59) | 100 (93-100) |
| Model 6 (Pattern 4 versus Pattern 1/2/3) |  | 4 | 0 | 3 | 67 | 57 (18-90) | 100 (95-100) | 100 (40-100) | 96 (88-99) |
| **Tram track sign** |  |  |  |  |  |  |  |  |  |
| Present at 3 months | 33 (48)† | 1 | 32 | 5 | 31 | 17 (0.4-64) | 49 (36-62) | 3 (0.1-16) | 86 (71-95) |
| Present at 6 months | 39 (57)† | 1 | 38 | 5 | 25 | 17 (0.4-64) | 40 (28-53) | 3 (0.1-14) | 83 (65-94) |
|  |  |  |  |  |  |  |  |  |  |

TRG: Tumour regression grade. MRI: magnetic resonance imaging. TP: true positive. FP: false positive. FN: false negative. TN: true negative.

CI: confidence interval. PPV: positive predictive value. NPV: negative predictive value. *Compared with baseline MRI scans. †Based on 69 canal tumours.
