# Supplementary material for: The assessment of local response using magnetic resonance imaging at 3- and 6-month post chemoradiotherapy in patients with anal cancer
Source: Eur Radiol. 2016 Apr 18;27(2):607–17. doi: 10.1007/s00330-016-4337-z (PMC5209434; doi:10.1007/s00330-016-4337-z)
Supplement: Supplementary file 3 — (DOC 85 kb) [file 330_2016_4337_MOESM3_ESM.doc]

**Table 3** Patient, tumour and imaging characteristics and prognostic modelling for disease-free survival (DFS)

|  |  |  | **Prognostic (Cox) modelling**  **Hazard ratios (95% CIs)** | | | |
| --- | --- | --- | --- | --- | --- | --- |
| **Characteristic** | **n** | **3-year DFS%** | Univariate |  | Multivariate |  |
| All | 74 | 72 |  |  |  |  |
| **Age category** |  |  |  |  |  |  |
| < 60 years (referent) | 33 | 70 | 1.00 |  |  |  |
| 60 to 74 years | 32 | 69 | 0.97 (0.40-2.33) | 0.95 |  |  |
| ≥ 75 years | 9 | 88 | 1.06 (0.29-3.86) | 0.93 |  |  |
| **Gender** |  |  |  |  |  |  |
| Male (referent) | 34 | 73 | 1.00 |  |  |  |
| Female | 30 | 70 | 1.11 (0.49-2.53) | 0.80 |  |  |
| **Performance status** |  |  |  |  |  |  |
| 0 (referent) | 32 | 75 | 1.00 |  | 1.00 |  |
| 1 | 26 | 73 | 1.02 (0.38-2.76) | 0.95 | 1.06 (0.34-3.23) | 0.92 |
| 2 | 5 | 20 | 5.86 (1.77-19.4) | 0.004 | 8.17 (1.83-36.6) | 0.006 |
| Missing | 11 | 82 | 0.93 (0.25-3.44) | 0.92 |  |  |
| **T stage** |  |  |  |  |  |  |
| T1/T2 (referent) | 54 | 81 | 1.00 |  | 1.00 |  |
| T3 | 10 | 60 | 2.90 (1.01-8.39) | 0.05 | 3.36 (0.88-12.9) | 0.08 |
| T4 | 10 | 30 | 5.00 (1.92-12.9) | 0.001 | 3.60 (0.81-15.9) | 0.09 |
| **N stage** |  |  |  |  |  |  |
| Negative (referent) | 38 | 76 | 1.00 |  |  |  |
| Positive | 36 | 67 | 1.27 (0.56-2.88) | 0.57 |  |  |
| **Circumferential involvement** |  |  |  |  |  |  |
| < 50% circumferential | 39 | 82 | 1.00 |  | 1.00 |  |
| ≥ 50% circumferential | 17 | 70 | 1.88 (0.66-5.44) | 0.24 | 1.20 (0.27-5.26) | 0.65 |
| Entire circumference | 18 | 50 | 3.21 (1.24-8.34) | 0.02 | 2.07 (0.56-7.62) | 0.28 |
| **Tumour extension into lower rectum** |  |  |  |  |  |  |
| No | 42 | 74 | 1.00 |  |  |  |
| Yes | 32 | 69 | 1.14 (0.50-2.59) | 0.75 |  |  |
| **Trans-sphincteric extension** |  |  |  |  |  |  |
| No | 61 | 77 | 1.00 |  |  |  |
| Yes | 13 | 45 | 2.47 (1.01-6.03) | 0.05 | Not in final model* | |
| **Involvement of adjacent organs** |  |  |  |  |  |  |
| No | 64 | 78 | 1.00 |  |  |  |
| Yes | 10 | 30 | 3.95 (1.62-9.67) | 0.003 | Not in final model* | |
| **TRG category at 3 months** |  |  |  |  |  |  |
| TRG 1/2 | 26 | 77 | 1.00 |  | 1.00 |  |
| TRG 3 | 43 | 72 | 1.26 (0.50-3.15) | 0.62 | 1.30 (0.42-3.99) | 0.65 |
| TRG 4/5 | 5 | 40 | 4.72 (1.22-18.4) | 0.03 | 2.18 (0.40-11.9) | 0.37 |
| **TRG category at 6 months** |  |  |  |  |  |  |
| TRG 1/2 | 51 | 78 | 1.00 |  |  |  |
| TRG 3 | 19 | 68 | 1.39 (0.53-3.67) | 0.50 |  |  |
| TRG 4/5 | 4 | 0 | Not estimable | |  |  |
| **TRG temporal pattern** |  |  |  |  |  |  |
| Pattern 1: TRG 1/2 at 3 months | 26 | 77 | 1.00 |  |  |  |
| Pattern 2: TRG 3 at 3 months → TRG 2 at 6 months | 28 | 79 | 0.99 (0.35-2.82) | 0.98 |  |  |
| Pattern 3: TRG 3/4 at 3 months → TRG 3 at 6 months | 16 | 69 | 1.39 (0.44-4.39) | 0.57 |  |  |
| Pattern 4: TRG 3/4 at 3 months → TRG 4/5 at 6 months | 4 | 0 | Not estimable† | |  |  |
| **Tram track sign at 3 months** |  |  |  |  |  |  |
| No | 41 | 71 | 1.00 |  |  |  |
| Yes | 33 | 73 | 0.93 (0.41-2.12) | 0.86 |  |  |
| **Tram track sign at 6 months** |  |  |  |  |  |  |
| No | 35 | 69 | 1.00 |  |  |  |
| Yes | 39 | 74 | 0.78 (0.34-1.76) | 0.55 |  |  |
|  |  |  |  |  |  |  |

CI: confidence interval. TRG: tumour regression grade. Performance status by WHO criteria.

*Not entered in final model because of collinearity problems with T-stage.

†Not estimable due to 100% events i.e. zero survival.
