# Supplementary figures and images for: The assessment of local response using magnetic resonance imaging at 3- and 6-month post chemoradiotherapy in patients with anal cancer
Source: Eur Radiol. 2016 Apr 18;27(2):607–17. doi: 10.1007/s00330-016-4337-z (PMC5209434; doi:10.1007/s00330-016-4337-z)

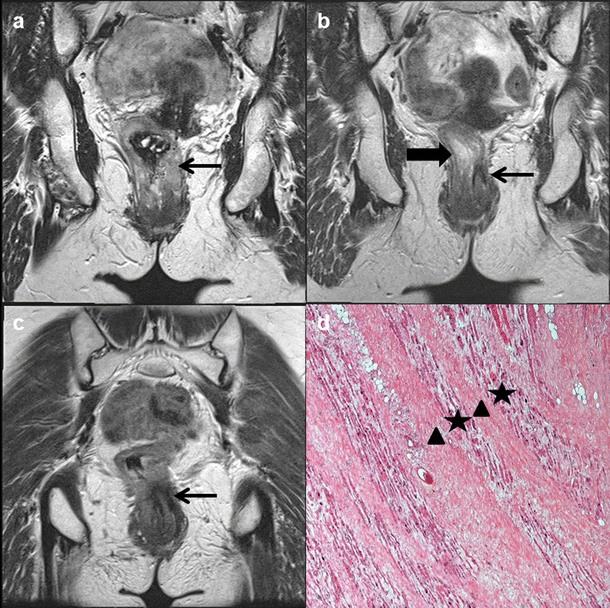

Supplement: Supplementary file 4 — High resolution coronal T2-weighted images (a-c). Staging MRI (a), showing an intermediate signal intensity tumour (arrow) in the anal canal involving the left internal sphincter. The 3-month (b) and 6-month post-CRT MRI (c), showing parallel linear low signal at the inner and outer margin of the left internal sphincter, at the site of the original tumour in keeping with a tram track sign (arrows) with no suspicious residual intermediate signal. Please note high signal mucosal oedema (block arrow) at the anorectal junction on the 3-month post CRT MRI. Photomicrograph with Haematoxylin and Eosin (H & E) stain and 20X magnification of a section of the outer portion of the internal anal sphincter (d), showing bands of stromal fibrosis in response to prior radiotherapy (triangle shapes), alternating with bands of residual atrophic skeletal muscle bundles (star shapes) which accounts for the tram track appearance. No residual invasive tumour seen. These histological features correlate with TRG 2 on post-CRT MRI (GIF 282 kb) [file 330_2016_4337_Fig7_ESM.gif]

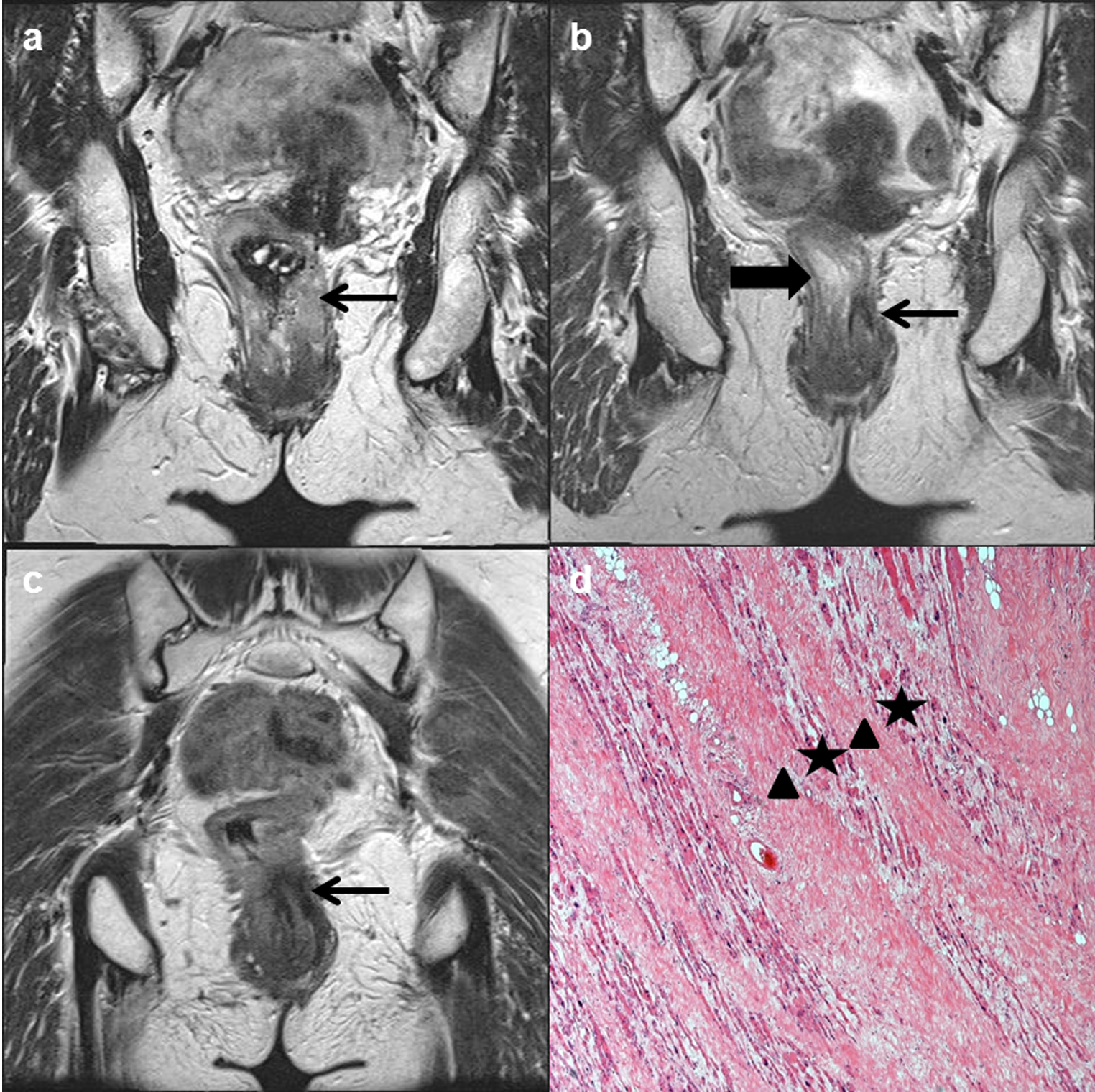

Supplement: Supplementary file 5 — High resolution image (TIF 3981 kb) [file 330_2016_4337_MOESM4_ESM.tif]

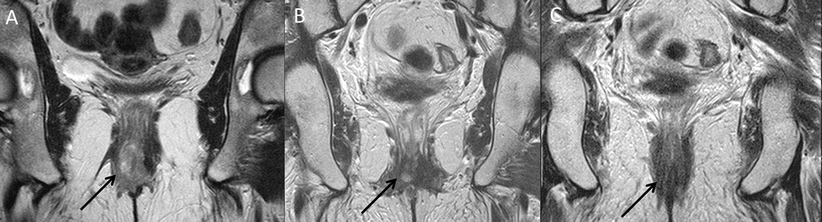

Supplement: Supplementary file 6 — High resolution coronal T2-weighted images (a-c). Baseline MRI (a), showing an intermediate signal intensity tumour (arrow) in the anal canal. The 3-month post CRT MRI (b) shows response to treatment but with suspicious intermediate signal at original tumour site (arrow), TRG score 4. EUA biopsy performed was however negative for disease. Note extensive high signal post treatment oedema at the anorectal junction. The 6-month post CRT MRI (c), now shows improvement in appearances with predominantly low signal change (arrow) downgrading the TRG score to 2 (GIF 98 kb) [file 330_2016_4337_Fig8_ESM.gif]

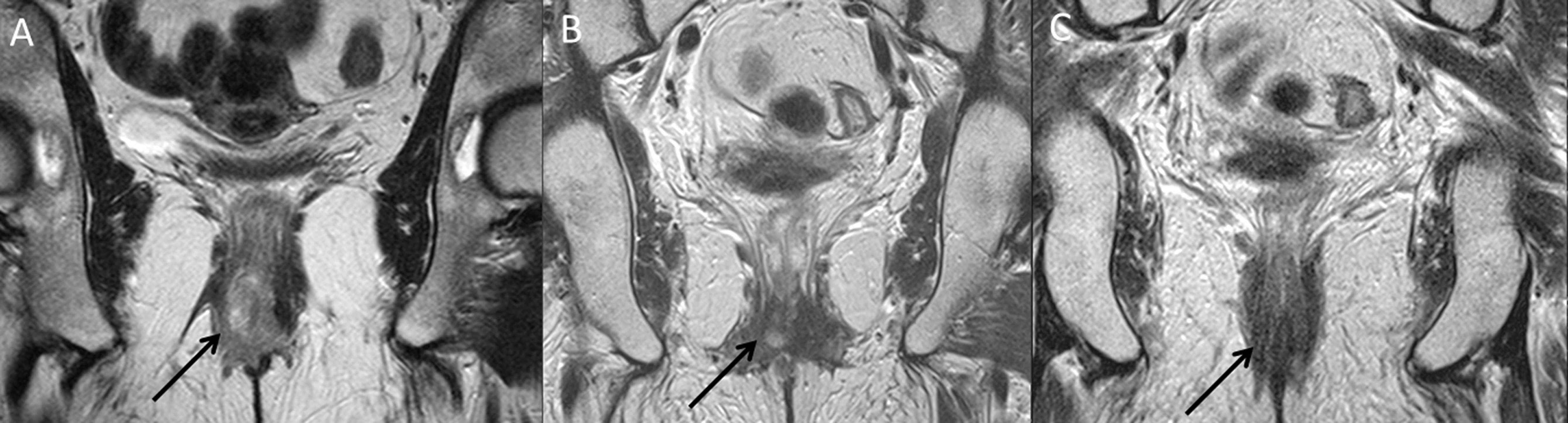

Supplement: Supplementary file 7 — High resolution image (TIF 4493 kb) [file 330_2016_4337_MOESM5_ESM.tif]
